# Protective Role of Platelets in Myocardial Infarction and Ischemia/Reperfusion Injury

**DOI:** 10.1155/2021/5545416

**Published:** 2021-05-24

**Authors:** Kornela Hałucha, Alina Rak-Pasikowska, Iwona Bil-Lula

**Affiliations:** ^1^Division of Clinical Chemistry and Laboratory Hematology, Department of Medical Laboratory Diagnostics, Faculty of Pharmacy, Wroclaw Medical University, Borowska 211 A, 50-556, Wroclaw, Poland; ^2^Lower Silesian Oncology Center, Hirszfelda 12, 53-413, Wroclaw, Poland

## Abstract

Thrombotic occlusion of the coronary artery is a key component in the pathogenesis of myocardial ischemia and myocardial infarction (MI). The standard therapy for ischemia is revascularization and restoration of blood flow to previously ischemic myocardium. Paradoxically, reperfusion may result in further tissue damage called ischemia/reperfusion injury (IRI). Platelets play a major role in the pathogenesis of MI and IRI, since they contribute to the thrombus and microthrombi formation, inflammation, release of immunomodulatory mediators, and vasoconstrictive molecules. Antiplatelet therapies have proven efficacy in the prevention of thrombosis and play a protective role in cardiac IRI. Beyond the deterioration effect of platelets in MI and IRI, in the 90s the first reports on a protective effect of molecules released from platelets during MI appeared. However, the role of platelets in cardioprotection is still poorly understood. This review describes the involvement of platelets in MI, IRI, and inflammation. It mainly focuses on the protective role of platelets in MI and IRI. Platelets are involved in cardioprotection based on platelet-releasing molecules and antiplatelet therapy, apart from antiaggregatory effects. Additionally, the use of platelet-derived microparticles as possible markers of MI, with and without comorbidities, and their role in cardioprotection are discussed. This review is aimed at illustrating the present knowledge on the role of platelets in MI and IRI, especially in a context of cardioprotection.

## 1. Introduction

Activated platelets play an important role in cardiovascular diseases especially in myocardial infarction (MI). They participate in the thrombus formation and microembolization, contributing to coronary artery occlusion. In addition, in many cardiovascular diseases, such as heart failure [1], coronary heart disease [2], and MI [3], an inflammatory component involving platelets has been observed. Besides, the detrimental effect of platelets in MI, the cardioprotective role of platelets has also been shown. The first reports on protective effects of platelets in MI appeared in the 1990s and were mainly focused on the protective effects of molecules released from the platelets during their activation and aggregation [4–6]. However, the cardioprotective properties of platelets are still poorly known.

In this paper, we present several aspects of the platelets interactions. The first one is the role of platelets in the pathology of MI, ischemia/reperfusion injury (IRI), and inflammation, particularly in the aspect of platelet-neutrophil and platelet-endothelium interactions. The second one is the protective role of platelets molecules in MI and IRI, as well as platelet-mediated mechanisms of cardioprotection. The importance of platelet microvesicles (PMVs) as possible biomarkers of thrombosis and cardiovascular events and the protective effect of PMVs are discussed. Finally, cardioprotective effects of antiplatelet therapy apart from antiaggregatory effect are discussed.

## 2. Platelets in Myocardial Infarction and Ischemia/Reperfusion Injury

The major role of platelets in MI is a thrombotic occlusion of the macroscopic and microscopic coronary vessels. The denudation of the vessel wall, as a result of unstable plaque rupture or intravascular intervention, leads to exposure of subendothelial matrix. The interaction of platelets with damaged vascular wall results in adhesion, activation, and aggregation of platelets. The subendothelial extracellular matrix is composed primarily of collagen fibrils, proteoglycans, glycoproteins (GP), and water [7]. Von Willebrand factor (vWF) secreted from the endothelial cells partially adheres to the subendothelial matrix collagen, while the majority is released into the lumen [8]. The adhesion of platelets to the damaged vessel wall is possible due to the presence of several receptors on platelet surface. The complex of GPIb-IX-V and integrin *α*IIb*β*3 (GPIIb/IIIa) binds to vWF immobilized by collagen, while GPVI and integrin *α*2*β*1 (VLA-2, GPIa/IIa) bind directly to subendothelial collagen [9–11]. The adhesion of platelets to the subendothelial layer triggers the platelets activation, which results in platelets morphology changes and the expression of platelets receptors, leading to alterations in the platelets phenotype [12]. GPVI has been established as a crucial molecule for platelets-vessel wall interactions, due to the initial role in adhesion, activation, and aggregation of platelets at the site of vascular damage [13]. Activated platelets release soluble mediators like thromboxane A2 (TXA2) and adenosine diphosphate (ADP) and generates thrombin. TXA2 is formed de novo from arachidonic acid (AA) through the activation of platelet cyclooxygenase- (COX-) 1. ADP, TXA2, and thrombin contribute to the formation of a blood clot by the stimulation of circulating platelets for activation and aggregation in the process named platelet recruitment [14, 15]. Furthermore, TXA2 has vasoconstrictive properties, which slows down blood flow and contributes to the thrombus formation [16]. Fibrinogen attaches to activated GPIIb/IIIa on platelets surface and acts as a bridge between them and thrombus formation, leading to vessel closure and MI [17]. Platelets are involved in the thrombotic occlusion of an epicardial coronary artery, due to eroded atherosclerotic plaque. They are responsible for microembolization by atherothrombotic platelet-rich aggregates formation. Going further, microembolization leads to decreased regional coronary blood flow and then transient myocardial ischemia [18]. Release of serotonin, TXA2, and free radicals by platelets in embolized vessels leads to vasoconstriction. Further roles of platelets in MI include enhanced intravasal thrombus formation in the microcirculation and mediation in inflammatory processes in the ischemic myocardium [18]. Besides the detrimental role of platelets in MI, the release of platelets content has been shown to have beneficial effects on the integrity of the coronary endothelium [19].

Inhibition of GPVI receptor was shown to be protective. Using anti-GPVI antibody (anti-GPVI JAQ1), Pachel et al. [20] achieved a significant reduction in INF/AAR (infarct size per area at risk) after 30 minutes of ischemia and 24 hours of reperfusion, increase in myocardial microperfusion after ischemia/reperfusion (I/R), and reduction in the number of neutrophils in the myocardium. Anti-GPVI JAQ1 antibody, which induces irreversible downregulation of the GPVI receptor on the circulating platelets surface, leads to irreversible depletion of GPVI and abnormal cell response for collagen. It has been proposed that the anti-GPVI JAQ1 may become an alternative to Revacept, an anticoagulant drug that is a GPVI-Fc dimeric fusion protein consisting of the extracellular platelet GPVI domain for collagen and the human Fc fragment. Anti-GPVI JAQ1 reduced platelet adhesion and aggregation mediated by collagen at the site of vascular damage. As a consequence, it reduced the infarct size [21, 22]. Using different GPVI antibodies (OM2), the reduction of infarct size in macaque monkey hearts has also been obtained [23].

### 2.1. Role of Platelets in IRI Ends Deleterious Effect of Platelets

The standard treatment for myocardial ischemia is revascularization and restoration of blood flow. Paradoxically, the restoration of blood flow to the previously ischemic myocardium may cause further tissue damage, called IRI [24]. The pathophysiology of IRI involves the ion accumulation, dissipation of mitochondrial membrane potential, formation of reactive oxygen species (ROS), disruption of nitric oxide (NO) metabolism, endothelial dysfunction, platelets activation, microembolization, immune activation, apoptosis, and autophagy [25]. The platelets get activated at an early stage of reperfusion, and then, platelets' accumulation occurs within the ischemic myocardium [26]. Platelets are involved in the pathology of IRI by several mechanisms. These mechanisms include platelets aggregation and microthrombi formation, platelet-leukocyte aggregation, release of exosomes and vasoconstrictors, PMVs and apoptotic body formation, and spinal afferent nerve activation [27]. Platelets aggregation and formation of microthrombi in small cardiac vessels and capillaries exert cardiac tissue damage [27]. It is known that platelets activation lead to release of granules content [28]. The platelet degranulation may lead to platelet-leukocyte and platelet-endothelium interactions and then development of an inflammatory response. Formation of platelet-leukocyte aggregates is mediated by interaction of P-selectin with P-selectin glycoprotein ligand-1 (PSGL-1) and platelet integrins with leukocyte macrophage-1 antigen (MAC-1), discussed in detail later [29]. Platelet-leukocyte aggregates promote further leukocytes infiltration into the I/R tissue [27]. There are several reports showing that reduction of platelet-neutrophils interactions may have a protective effect during IRI [30]. Recent studies have shown that platelet-derived serotonin enhanced inflammation in myocardial IRI due to induced neutrophils degranulation. The authors concluded that serotonin serves as a potent therapeutic target in neutrophil-dependent thromboinflammation during myocardial IRI [31]. Moreover, microvesicles (MVs), apoptotic bodies, and exosomes have the potential to enhance inflammation within the I/R myocardium. Release of TXA2, by activated platelets, also leads to endothelial dysfunction [27].

Role of platelets in the pathogenesis of IRI has also been associated with generation of ROS. In the studies of guinea pig hearts exposed to I/R, administration of activated platelets at early stage of reperfusion led to reduction of recovery of external heart work (REHW). Cardiodepressive effect of platelets was mediated by ROS released from platelets [32]. Detrimental effects of activated platelets on left ventricular (LV) function following cardiac IRI have been shown [33]. Alternatively, Muessig et al. demonstrated the protective effect of platelet-rich plasma (PRP) on LV recovery and function after IRI [34].

Mirabet et al. showed that different effects of platelets during I/R depend on the platelets activation status. They showed that transient coronary occlusion caused increased platelet accumulation and activation and further myocardial damage, since platelets obtained 10 minutes after reperfusion increased lactate dehydrogenase (LDH) release from isolated hearts subjected to I/R [33]. Fucoidan, a selectin blocker, has been shown to reduce myocardial necrosis after reperfusion in pigs and protect isolated I/R rat hearts in the presence of platelets. The authors suggested that selectin is involved in platelet deposition in reperfused cardiac microvessels [30]. Activated platelets obtained from patients with acute MI (AMI) increased myocardial injury in isolated rat hearts subjected to I/R, compared with platelets from control donors. This effect was attenuated by inhibition of P2Y12 and GPIIb/IIIa [35]. Recently, Targ-CD39 (fusion protein that specifically binds to GPIIb/IIIa) was shown to reduce infarct size and increase neovascularization in I/R mouse model. Targ-CD39 prevented thrombus formation and vessel occlusion. The authors suggested that Targ-CD39 has the potential to attenuate microthrombi formation in small cardiac vessels and capillaries after reperfusion. This indicates the participation of GPIIb/IIIa in these pathological processes [36]. In addition to platelet membrane proteins, such as GPVI, P2Y12 receptor, GPIIb/IIIa, and P-selectin, mediators released from platelets may also reinforce the negative effects in MI hearts. This includes ROS, serotonin, and high quantities of platelet activating factor (PAF) (discussed in detail later) [28].

### 2.2. Platelets Trigger Inflammation

**Figure 1:**
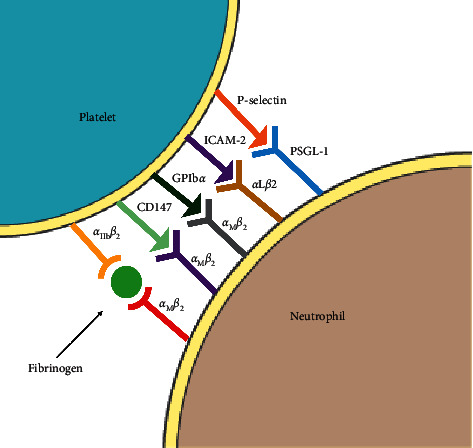
Overview of the most important platelets selectins and integrins related to platelets and neutrophils adhesion in MI. PSGL-1 receptor binding is the first interaction between platelets and neutrophils. This interaction is stabilized by secondary adhesion interactions including directly binding GPIb*α* and indirectly binding *α*IIb*β*3 of platelets with *α*M*β*2 of neutrophils. The importance of interactions of ICAM-2 with *α*L*β*2 and CD147 with *α*M*β*2 in platelet-neutrophil adhesion is still poorly understood. PSGL1: P-selectin glycoprotein ligand-1; ICAM2: intercellular adhesion molecule; MI: myocardial infarction. The server https://smart.servier.com was used to create a figure.

In MI, platelets play a role in initiating and modulating the inflammation due to the presence of adhesive molecules and receptors on their surface and the release of their immunomodulatory content [37]. The most important selectin, involved in inflammation, is P-selectin. When expressed on activated platelets surfaces, P-selectin is involved in direct platelet-neutrophil interactions [38]. The binding of P-selectin to the PSGL-1 neutrophil receptor is necessary to initiate interactions between platelets and neutrophils and the formation of platelet-neutrophil aggregates [29]. Alternatively, platelet-neutrophil aggregates were shown to exert protective effect in resolution of inflammation in the IRI [39, 40]. Several animal studies have shown a positive effect of P-selectin blocking in the development of cardiac IRI, manifested as less neutrophils infiltration or decreased platelets adhesion to neutrophils in the infarcted region [41, 42]. The surface adhesion molecules of platelets taking part in the immune response are mainly integrins, selectins, and adhesive molecules like platelets endothelial cell adhesion molecule-1 (PECAM-1) [43–45]. Meta-analysis provided by Sahebkar et al. suggested a protective role of the rare homozygous genotype of the rs1131012 single nucleotide polymorphism in the PECAM-1 gene against MI [46]. Integrins *α*IIb*β*3 and *α*2*β*1 take part in the aforementioned interaction of platelets with the intracellular matrix, endothelium, and leukocytes [47]. Another function of integrins is the ability to trigger outside-in signaling, which may result in activation and degranulation of platelets [48]. Activated neutrophil integrin *α*M*β*2 (MAC-1) binds with platelets directly by GPIb*α* and indirectly by integrin *α*IIb*β*3, through a “bridge” of fibrinogen. This enhances the interaction of platelets and neutrophils by stabilizing the platelets to neutrophils adhesion [49, 50]. Recently, new surface receptor of platelets CD147 was investigated as a binding partner for neutrophils MAC-1, in platelet-neutrophil adhesion, but the mechanism of this interaction is still unknown [51]. Platelet-neutrophil adhesion molecules are summarized in Figure 1.

Simultaneous perfusion with platelets and neutrophils has been shown to exacerbate cardiac contractile dysfunction in isolated heart model of IRI [52]. Alternatively, Seligman et al. showed that platelets and neutrophils coinfused in guinea pig hearts exposed to low-flow ischemia have no additive cardiodepressive effect than infused separately [53].

The dynamic reorganization of neutrophils domains and receptors provides interaction with activated platelets at early stages of inflammation [38]. During platelet-neutrophil interactions maresin 1 (MaR1), one of the proresolving mediators is amplified. MaR1 biosynthesis can be observed primarily in leukocytes but low amounts of MaR1 can also be produced in platelets [54]. MaR1 regulates platelet hemostatic functions and inflammation. MaR1 have also been shown to be organ protective in the model of acute lung injury [54, 55].

Platelets also enhanced chemotaxis of other inflammatory cells to the site of tissue damage, through platelet content secreted during activation and adhesion of platelets to the damaged vascular endothelium [56]. Moreover, PMVs, discussed in detail later, constituent the source of AA and induce monocytes adhesion to vascular endothelial cells [57].

### 2.3. Cardioprotective Effect of Platelets

#### 2.3.1. Direct Cardioprotective Effect of Platelets

Platelets have been shown to be involved in the regeneration of the heart muscle after MI. Platelets contain, and through the activation release, CXCL12 (stromal cell derived factor-1*α* (SDF-1*α*)). CXCL12 is a chemokine capable to initiate the progenitor cells migration to the site of damage leading to organs and tissues repair. CXCL12 recruits CD34+ cells and affects their proliferation and differentiation into different cell types, such as macrophages, foam cells, and endothelial cells [58, 59]. Increased platelet CXCL12 expression in MI patients has been shown to be associated with improved heart function and reduced infarct size [60]. The highest expression of SDF-1*α* was observed in the peri-infarction zone [61]. In patients with symptomatic coronary artery disease (CAD), expression of transforming growth factor beta 1 (TGF-*β*1) on the platelet surface correlated with the expression of SDF-1*α* and SDF-1*α* receptors: CXCR4 and CXCR7. TGF-*β*1 expression increased significantly after platelet stimulation with recombinant SDF-1*α* in vitro [62]. TGF-*β*1 is released from platelets during activation. It is involved in the suppression of inflammatory response [63], heart repair, and remodeling [64]. Higher TGF-*β*1 expression on platelet surface was observed in patients with acute coronary syndrome (ACS), compared with patients with stable CAD. In addition, low TGF-*β*1 platelets expression was associated with increased mortality and incidence of ST-segment elevation MI (STEMI) [65].

**Figure 2:**
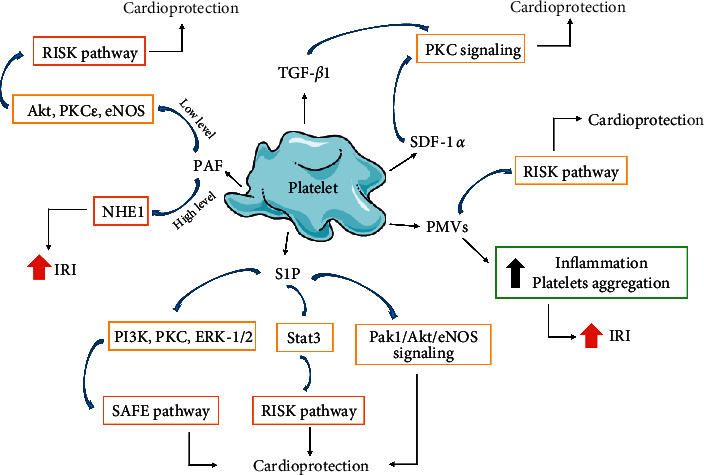
Cardioprotective role of platelets in ischemia/reperfusion injury (IRI). S1P can exert cardioprotection by activating RISK and SAFE-pathway, including activating kinases PI3 K, PKC, ERK-1/2, and Stat3, respectively. Moreover, S1P triggers cardioprotection by activation of Pak1/Akt/NOS3 signaling. Cardioprotective effect for TGF-*β*1 and SDF1-*α* is based on PKC signaling activation. PAF and PMVs have a dual effect on cardiac IRI. In the case of PAF, this effect depends on the level of PAF. RISK: reperfusion injury salvage kinases, SAFE: survivor activating factor enhancement, Akt: protein kinase B, ERK: extracellular signal-regulated kinase, Pak1: P21-activated kinase; PKC: protein kinase C, PI3 K: phosphatidylinositol 3-kinase, Stat3: signal transducer and activator of transcription 3, eNOS: endothelial nitric oxide synthase, NHE1: Na(+)/H(+) exchanger isoform 1, S1P: sphinosine-1-phosphate, SDF-1*α*: stromal cell derived factor-1 alpha, TGF-*β*1: transforming growth factor beta 1. The blue arrows indicate activation of the signaling path shown. The server https://smart.servier.com was used to create a figure.

Studies on isolated rat hearts showed that platelets reduced the heart dysfunction due to I/R. The protective effect of platelets was evident in the presence of a much lower platelet count (10^5^ cells/mL) than that found in the physiological conditions (5-10 × 10^8^ cells/mL) in rats. A conclusion has been drawn that platelets have a strong protective potential to cardiomyocytes against ischemia [4]. The protective effect of platelets on the heart subjected to IRI is attributed to molecules released from the platelets during activation and aggregation. Many studies have shown that the supernatant formed after platelet aggregation, infused into rat hearts, has cardioprotective properties. The cardioprotective effect of platelets is mainly attributed to the release of adenine nucleotides, TXA2, and serotonin, which induce the release of endothelial NO that exerts a cardioprotective effect [6, 66, 67]. The protective effect of platelets is partially attributed to the substances released from the alpha granules, since the cardioprotective effect of platelets is present regardless of the contribution of dense platelet granules [66]. TGF-*β*1, released from the alpha granules, showed a protective effect on the rat myocardium and reduced apoptosis of cardiomyocytes due to hypoxia and reoxygenation [5, 68]. In addition, SDF1-*α*, released from the alpha granules, showed a cardioprotective effect, resulting in the delay of cardiomyocytes death [66]. Receptors for both TGF and SDF, TGF*β*R1 and CXCR4, respectively, are found on cardiomyocytes [69, 70]. TGF-*β*1 and SDF1-*α* initiate protein kinase C (PKC) signaling in cardiomyocytes as a prosurvival mechanism during ischemia (Figure 2) [66].

A cardioprotective effect on the heart in MI has also been demonstrated for sphingosine-1-phosphate (S1P). Platelets contain sphingosine kinase, which converts membrane sphingosine into S1P. S1P is stored in platelets and released in large amounts during platelet activation, thrombus formation, and inflammatory processes, including MI [71]. S1P contributes to regular thrombopoiesis by acting on megakaryocytes, while the effect of S1P on platelets may be manifested by both pro- and antiaggregatory effects through G-protein coupled receptors (GPCRs) [72, 73]. In the studies on isolated cardiomyocytes and perfused rats hearts ex vivo, the cardioprotective effect of S1P has also been demonstrated [74, 75]. An increase in cardiomyocytes viability under hypoxic conditions and a reduction in heart infarct size after I/R were observed. S1P also mediated in the beneficial effects of ischemic preconditioning (IPC) and postconditioning of hearts undergoing I/R [76, 77]. There are three receptors for S1P in the heart: S1P1, S1P2, and S1P3. However, it is postulated that S1P reduces damage during I/R by stimulation of S1P2 and S1P3 with G-protein coupled receptors, which leads to the activation of protein kinase B (Akt), signal transducer and activator of transcription 3 (Stat3) kinases, reperfusion injury salvage kinases (RISK), and survivor activating factor enhancement (SAFE) pathway [76, 78]. Likewise, activation of P21-activated kinase (Pak1)/Akt/endothelial nitric oxide synthase (eNOS) signaling through S1P triggers cardioprotection (Figure 2) [79]. S1P and platelets have been shown to be essential factors in cangrelor-induced cardioprotection, one of the inhibitors of the P2Y12 receptor [80].

Cardioprotection of platelet S1P is mediated by activation of RISK pathway (PI3 K, ERK-1/2, and PKC) [81]. In type 2 diabetes mellitus (T2DM), hyperreactivity of platelets was observed. This hyperreactivity was associated with prothrombotic state [81, 82]. T2DM platelets released less S1P than platelets from healthy participants. The authors suggested that the altered redox conditions of diabetes platelets dysregulated S1P release and abolished cardioprotection mediated by RISK pathway [81].

#### 2.3.2. Indirect Cardioprotective Effect of Platelets

PAF is a phosphoglyceride, produced by platelets, leukocytes, and endothelium cells [83, 84]. PAF, as a paracrine and autocrine mediator, can act on a variety of cell types including cardiomyocytes, platelets, and endothelium cells. It acts through specific receptors (PAFRs) present on the surface of cardiomyocytes [85]. Depending on its concentration, PAF can induce different effects during cardiac IRI. Low levels of PAF (in pmol/L range) are not able to alter myocardial contractility; however, it can exert IPC-like cardioprotection by activating RISK signaling kinases, including PKC, Akt, and eNOS [85, 86]. Unfortunately, high doses of PAF (1-10 nmol/L) had a negative effect on the coronary and cardiac functions and exhibited a strong arrhythmogenic effect (Figure 2) [85]. The use of a PAF receptor antagonist prior to reperfusion can significantly reduce the infarct size, not only by direct inhibition of PAR receptor but also by an antiplatelet effect [87, 88]. High doses of PAF seem to have a direct cardiodepressive effect. PMVs, discussed in detail in next section, seem to have an indirect cardioprotective effect. Description of cardioprotection mediated by platelet agents is summarized in Table 1.

Mitophagy has been described as one of the platelet-mediated cardioprotection mechanisms in IRI. Mitophagy is a form of autophagy involving the degradation of excessive or damaged mitochondria, as an early mitochondrial response to hypoxia [90]. Mitophagy not only regulates the quality and quantity of mitochondria but also affects the activation of platelets during cardiac IRI [91]. Platelet mitochondrial dysfunction leads to reduced ATP production, impaired calcium buffering, and generation of ROS [92]. As platelet activation is an energy-dependent process, fully functional mitochondria are required [93]. Thus, dysfunctional platelets' mitochondria can inhibit platelets activation. Platelet mitophagy plays a dual role in cardiac IRI. In the early stages, only damaged platelet mitochondria are removed [91, 94]. Functional mitochondria enable the activation of platelets and their adherence to the site of injury and cause MI. In the later phase of I/R, when the oxygen level becomes low, hypoxic mitophagy results in a significant elimination of mitochondria (possibly also undamaged ones), resulting in decreased platelet activation. Under hypoxic conditions, FUN14 domain containing 1 (FUNDC-1) and autophagy related 5- (ATG5-) dependent mitophagy are present, the effect of which is an intense degradation of platelet mitochondria [91]. Mitophagy prevents excessive platelet activation and myocardial injury through negative self-regulation mechanism. Thus, it may serve as a possible protective mechanism against IRI [91, 95]. Platelet mitophagy seems to be a key element in IPC. IPC activates FUNDC-1-mediated platelet mitophagy. This leads to extensive degradation of mitochondria, reduction of platelets activation, and, consequently, a reduction in the myocardial infarct size and maintenance of heart function [91]. The involvement of mitophagy in the activation of platelets and then in IRI may be an interesting subject of research, especially in searching for new molecules that can regulate mitophagy pathways. Melatonin has been proposed as a molecule important for platelet mitophagy in FUNDC-1-dependent pathway [96].

### 2.4. PMVs in Myocardial Infarction and Ischemia/Reperfusion Injury

Extracellular vesicles (EVs) are membranous vesicles, which carry bioactive molecules. EVs are mainly divided into exosomes, MVs, and apoptotic bodies [97]. As a result of activation, platelets release PMVs, also known as microparticles. MVs are membrane fragments, 0.1 to 1.0 µm in diameter, released from many cells, such as platelets, endothelium cells [98], leukocytes [99], or erythrocytes [100], due to their activation or apoptosis. MVs carry proteins, messenger RNA (mRNAs), microRNA (miRNAs), and lipids [101]. MVs can both contribute to the formation of pathological conditions and be their effect [102]. MVs contain phospholipids and membrane fragments from the cell they originate, enabling differentiation between them by the origin. PMVs present surface proteins characteristic for the platelets, such as CD62P, CD63, and CD41. PMVs have prothrombotic properties, modulate intercellular interactions, and mediate inflammatory processes [103, 104]. In normal conditions, PMVs constitute the majority of MVs concentration. In ACS, including MI, PMV levels increase, although they appear to return to previous level at the disease stabilization [98]. Many studies support the use of PMVs as potential markers for MI and IRI in the future. Jung et al. showed that the level of circulating PMVs (CD31+/CD42+) correlates with the degree of ischemia and thrombosis in patients with STEMI [105]. Proteomic studies of MVs in STEMI patients established that, beside inflammation, PMVs are involved in excessive platelet aggregation, responsible for thrombosis [106]. PMVs were proposed as a marker of progressing thrombosis in STEMI patients. PMVs may have a role in the pathogenesis of microvascular obstruction [107]. Additionally, PMVs can potentially be used as biomarkers of increased risk of new cardiovascular events [108]. In addition, PMVs are involved in cardioprotective effect of remote ischemia preconditioning (RIPC) in cardiac IRI. MVs isolated from RIPC rats have been shown to reduce infarct area and ameliorate LV function in IRI rats [109]. Moreover, PMVs induce angiogenesis, and this effect is mediated by cytokines, vascular endothelial growth factor (VEGF), basic fibroblast growth factor (bFGF), and platelet-derived growth factor (PDGF). PMVs have potential to contribute to revascularization after chronic ischemia, since injection of PMVs into the ischemic myocardium provoked increased number of functioning blood vessels by activation of VEGF-mediated RISK pathway [89]. Liu et al. showed protective role of circulating MVs derived from ischemic preconditioning (IPC-MVs) on myocardial IRI in rats. PMVs were increased in circulation of IPC rats. IPC-MVs were shown to alleviate damage of myocardium and restored cardiac function after IRI [110]. These effects were achieved through endoplasmic reticulum stress- (ERS-) dependent attenuation of cell death induced by ERS (inhibition of caspases 3 and 12) [110]. Additionally, in cardiovascular diseases such as ACS, heart failure, or MI, platelet activation leads to changes in their protein composition [111]. In cardiovascular diseases, inflammatory proteins like S100A8 were identified in platelets. Cheow et al. identified six upregulated proteins in plasma EVs of MI patients compared to patients with stable angina. Two of them, platelet glycoprotein Ib alpha chain and platelet basic protein, are related to the role of platelets in inflammation and in platelets activation pathway [112]. The platelet proteome may provide specific markers for cardiovascular diseases. However, exploring these proteins requires further research [113, 114].

### 2.5. EVs as Biomarkers of Comorbidities Associated with Myocardial Infarction and Ischemia/Reperfusion Injury

EVs carry bioactive molecules, such as proteins, lipids, amino acids, mRNAs, and miRNAs. EVs cargo depends on many factors, including changes in the microenvironment such as oxygen content, inflammation, age, and gender [101, 115]. Going forward, risk factors and comorbidities associated with ischemic diseases, such as age, gender, and metabolic diseases, are linked with changes in circulating EVs; therefore, EVs can be useful biomarkers [116].

Several studies have shown that obesity affects the amount and composition of EVs [117–119]. Level of PMVs and endothelial MVs was elevated in obese woman compared with women of similar age with normal weight [117]. Increased level of PMVs was associated with excessive adipose tissue and metabolic state of adipocytes and platelets [115, 119]. Improvement in PMV levels has been reported in obese patients 12 months after gastrectomy or by caloric restriction and exercise [119, 120]. Rigamonti et al. showed reduced release of total EVs, including platelet EVs, 24 h after acute exercise. Additionally, higher postexercise release of platelet EVs was observed in females than in males [121]. Exercise, in turn, increased circulating EVs with a protective effect against IRI. Exercise-derived EVs protection was related with activation of ERK1/2 and heat shock protein 27 (HSP27) signaling in cardiac IRI. Therefore, exercise-derived EVs may serve as potent therapy against cardiac IRI [115, 118]. Platelet EVs formed during heavy exercises stimulated human endothelial cells by enhancing angiogenesis and proliferation in vitro [122]. Penna et al. showed that the inflammatory microenvironment containing IL-3 prevents cardioprotection mediated by endothelial cell- (EC-) derived EVs by changing EVs cargo [123].

An increase in PMVs level was found in patients with hypertension. PMVs were correlated with the level of systolic and diastolic blood pressures [124]. Penna et al. suggested that sex-dependent hormonal modulations may affect changes in EVs in pathological conditions. Therefore, in the context of EV as new biomarkers of ischemia/reperfusion and comorbidities, further research needs to paid more attention to gender influence [115].

Increased levels of PMVs were also shown in metabolic syndrome [125]. MVs isolated from plasma of metabolic syndrome patients have been identified as carriers of macrophage migration inhibitory factor (MIF). Since MIF is involved in the development of insulin resistance, associated with diabetes and obesity, and MV-associated MIF triggered rapid ERK1/2 activation in macrophages, the authors suggested that MIF pathway should be reconsidered in the context EV-associated form [126]. Hypercholesterolemia affected microRNA alterations in the rat myocardium, leading to subsequent dysfunction in the heart [127]. Transcriptome analysis may provide specific diagnostic markers of the heart affected by comorbidities [128].

Sabatier et al. showed elevated levels of PMVs in type 1 diabetic mellitus (T1DM) patients compared with age-matched control subjects. Moreover, in T1DM elevated EVs-associated procoagulant activity was correlated with hemoglobin A1c (HbA1c), suggesting that glucose imbalance and obesity may affect EVs cargo and biological effect of EVs [129]. In the study of children and adolescents with T1DM, Salem et al. showed increased levels of PMVs, especially in patients with microvascular complications. PMVs were related with obesity, hyperglycemia, inflammation, and microvascular complications in T1DM patients. The authors suggested that PMVs may serve as future markers of microvascular complications and subclinical atherosclerosis [130].

The increased PMVs concentration in patients with T2DM, compared with patients without diabetes, was independent of obesity. PMVs may contribute in pathogenesis of atherosclerosis and T2DM, since increased concentration of PMVs, which expressed fibrinogen and tissue factor, in subjects with T2DM was reported [131]. Aspirin therapy inhibited vascular wall cell activation and EVs and PMVs shedding in TDM1 and TDM2 [132]. Davidson et al. demonstrated impaired cardioprotecion ability of exosomes due to diabetes. Additionally, exosomes from a nondiabetic rat were able to protect cardiomyocytes from a diabetic rat, by ERK1/2 and HSP27 signaling, indicating that exosomes do not lose their cargo and functions in hyperglycemic environment and may serve as a potential therapy in diabetes [133].

### 2.6. Platelet NLRP3 as a Prognostic Factor in Patients with ACS

NOD-like receptor pyrin domain containing 3 (Nlrp3) inflammasome is one of the identified proinflammatory signaling pathways involved in cardiovascular disorders and comorbidities, for example, metabolic syndrome and obesity [134]. NLRP3 inflammasome is large multimeric protein complex, which interacts with an apoptosis-associated speck-like protein, activating caspase-1, which mediates the cleavage of inactive prointerleukin-1*β* and IL-18 into their active form [134]. NLRP3 inflamamsome may be involved in development of cardiovascular disorders associated with metabolic diseases. High-fat, high-fructose diet resulted in greater infarct size and lactic dehydrogenase release in mice hearts exposed to IRI compared with hearts of mice fed a standard diet. This diet induced abolished cardioprotection due to inhibition of protective signaling pathways: RISK/hypoxia inducible factor 2*α* was associated with overexpression of NLRP3 inflammasome [135]. In the study of T2DM rats, Birnbaum et al. showed adenosine-dependent additive cardioprotective effect of ticagrelor and rosuvastatin indicated as reduction of infarct size in cardiac IRI. Both ticagrelor and rosuvastatin reduced postinfarction activation of NLRP3 inflammasome [136]. Pharmacological inhibition of the NLRP3 inflammasome protected against IRI in rat hearts. This effect was linked with activation of RISK pathway and improvement in mitochondrial function [137].

NLRP3 regulates platelet spreading and clot retraction by a mechanism involving interleukin- (IL-) 1*β*. Qiao et al. suggested that NLRP3 plays a role in regulation of platelet function and thrombus formation, and new potential therapy strategies in treating inflammation-associated thrombosis may target NLRP3 or IL-1*β* [138]. Peng et al. showed higher expression of platelet NLPR3 in patients with ACS than in patients with stable angina pectoris (SAP). Additionally, among all patients with ACS, the highest expression of platelet NLPR3 was observed in patients with STEMI. Expression of platelet NLPR3 correlated with Gensini score and Global Acute Coronary Event (GRACE) score. Going forward, platelet NLPR3 expression should be considered as a novel potential prognostic factor for patients with ACS after PCI as high expression of platelet NLRP3 resulted in poorer prognosis after PCI [139]. The study of isolated rat hearts revealed that ticagrelor conditioning attenuated NLRP3 inflammasome complex formation through platelets. Cardioprotection evoked activation of RISK pathway and limitation of I/R-induced oxidative stress [140].

### 2.7. Cardioprotective Properties of Antiplatelet Therapy

The use of antagonists of platelets molecules with cardiodepresive effect or blocking of specific receptors resulted in cardioprotection. As in the aforementioned case, the use of anti-GPVI antibodies caused the suppression of platelet response to collagen [20]. As previously described, during the IRI, large doses of PAF are released and can negatively affect coronary and heart function. However, the use of a PAF receptor antagonist, prior to reperfusion, in a sheep and rabbit model reduced the infarct area and reperfusion injury [87, 88]. Current platelet-related therapies to prevent IRI include the use of COX (aspirin), P2Y12 receptor (clopidogrel and ticagrelor), GPIIb/IIIa, GPVI, and GPIb and P-selectin inhibitors. Apart from an effective antiaggregatory effects and indirect protection of the heart against IRI, a direct cardioprotective effect of these drugs has been proven [141–143]. Aspirin has been shown to prevent MI and have an anti-inflammatory effect [141, 144]. Aspirin attenuated endothelial dysfunction and modulated acetylcholine-induced peripheral vasodilation in patients with atherosclerosis, possibly via inhibition of COX-dependent vasoconstrictors [145]. However, in rabbit model of myocardial I/R, aspirin blocked AA-induced platelet aggregation but did not affect infarction [146]. The cardioprotective role of P2Y12-inhibitors, including clopidogrel, prasugrel, and ticagrelor, against IRI has been reported [146–149]. The protective effect of these drugs was initially associated only with the antiaggregatory effects. However, Yang et al. showed that cangrelor and clopidogrel protected hearts against infarction in rabbit model of myocardial I/R. This protective effect was independent of its antiaggregatory properties. The authors suggested that the protective effect of cangrelor was based on the same mechanism as ischemic postconditioning (IPOC). Individual blocking signaling elements, including adenosine A2B receptors, extracellular signal-regulated kinase (ERK), Akt, redox signaling, or mitochondrial ATP-sensitive potassium (KATP) channels, disrupted the protective effect of cangrelor [146]. A retrospective analysis by Rouille et al. showed a reduction of infarct size in patients with STEMI, as a result of direct clopidogrel effect [150]. The interaction of platelets with cangrelor seems to be necessary to trigger the cangrelor protective effect, since cangrelor did not reduce infarct size in thrombocytopenic rats subjected to 30 minutes of ischemia and subsequent two hours of reperfusion [80]. Penna et al. confirmed that interaction with platelets is necessary for the ticagrelor protection in rat hearts. Moreover, ticagrelor has the potential to induce the release of S1P and adenosine from platelets that trigger heart protection in vivo. S1P and adenosine were shown to reach the highest concentrations in rat hearts protected by ticagrelor. Both S1P and adenosine are involved in RISK pathway activation in the heart [140]. Additionally, ticagrelor was able to prevent endothelial cells against apoptosis due to hypoxia stress. The protective effect of ticagrelor was associated with the adenosine signaling pathway [151].

## 3. Conclusions

Although it is difficult to conclusively determine the effect of platelets on the heart in MI and IRI, there are growing lines of evidence that platelets molecules exert protective effect on the heart, triggering specific mechanisms of cardioprotection. Recently, the presented protective effects of cangrelor and ticagrelor, independent of the antiaggregatory effects, are strong evidence for the participation of platelets in the protection against cardiac IRI. Accurate investigation of the protective role of platelets in MI and IRI could lead to a more effective treatment. Additionally, the proposal to use PMVs as prognostic and predictive markers, and changes in platelets proteome may shed new light on the diagnostics of MI. Importantly, platelet exosomes may serve as useful biomarkers for MI in patients with comorbidities in the future. However, further research is needed to identify and determine the clinical utility of potential new platelet markers.

## Figures and Tables

**Table 1:** Description of cardioprotection mediated by platelet agents.

Agent	Cardiomyocytes receptor	Model of IRI	Mechanism of cardioprotection	Proposed cardioprotection influence^∗^	Ref.
SDF1*α*	CXCR4, CXCR7	Mice ventricular cardiomyocytes	Activation of PKC signaling	Direct	[66]
TGF-*β*1	TGF*β*R1	Mice ventricular cardiomyocytes	Activation of PKC signaling	Direct	[66]
S1P	S1P1, S1P2,S1P3	Rat ventricular cardiomyocytes, mice hearts	Receptor (S1P2, S1P3) mediated Pak1/Akt/eNOS signaling activation RISK and SAFE pathway activation	Direct	[78, 79]
PAF^∗∗^	PAFRs	Rat hearts	PI3 K and PKC activation, similar to IPC	Indirect	[86]
PMVs	NI	Rat aortic ring model, rats hearts	Activation of RISK pathway	Indirect	[89]

^∗^Cardioprotection effect proposed by authors of this review. ^∗∗^Low levels (in pmol/L range). IRI: ischemia/reperfusion injury, RISK: reperfusion injury salvage kinases, SAFE: survivor activating factor enhancement, Akt: protein kinase B, Pak1: P21-activated kinase; PKC: protein kinase C, PI3 K: phosphatidylinositol 3-kinase, eNOS: endothelial nitric oxide synthase, S1P: sphinosine-1-phosphate, SDF-1*α* : stromal cell derived factor-1 alpha, TGF-*β*1: transforming growth factor beta 1, IPC: ischemia preconditioning, and NI: not identified.

## Data Availability

Data are obtained from the references cited in the manuscript.

## References

[1] Stumpf C., Lehner C., Eskafi S. (2003). Enhanced levels of CD154 (CD40 ligand) on platelets in patients with chronic heart failure. *European Journal of Heart Failure*.

[2] Danesh J., Wheeler J. G., Hirschfield G. M. (2004). C-reactive protein and other circulating markers of inflammation in the prediction of coronary heart disease. *New England Journal of Medicine*.

[3] Kossmann H., Rischpler C., Hanus F. (2019). Monocyte-platelet aggregates affect local inflammation in patients with acute myocardial infarction. *International Journal of Cardiology*.

[4] Yang B., Mehta P., Mehta J. L. (1998). Platelet-mediated cardioprotective effect against ischemia-reperfusion injury in isolated rat hearts: role of platelet number and contribution of supernatant of aggregated platelets. *Journal of Cardiovascular Pharmacology and Therapeutics*.

[5] Yang B. C., Zander D. S., Mehta J. L. (1999). Hypoxia-reoxygenation-induced apoptosis in cultured adult rat myocytes and the protective effect of platelets and transforming growth factor-*β*1. *Journal of Pharmacology and Experimental Therapeutics*.

[6] Yang B. C., Mehta J. L. (1994). Platelet-derived adenosine contributes to the cardioprotective effects of platelets against ischemia-reperfusion injury in isolated rat heart. *Journal of Cardiovascular Pharmacology*.

[7] Fishman J. A., Ryan G. B., Karnovsky M. J. (1975). Endothelial regeneration in the rat carotid artery and the significance of endothelial denudation in the pathogenesis of myointimal thickening. *Laboratory Investigation; a Journal of Technical Methods and Pathology*.

[8] Stel H., Sakariassen K., de Groot P., van Mourik J., Sixma J. (1985). Von Willebrand factor in the vessel wall mediates platelet adherence. *Blood*.

[9] Savage B., Almus-Jacobs F., Ruggeri Z. M. (1998). Specific synergy of multiple substrate-receptor interactions in platelet thrombus formation under flow. *Cell*.

[10] Moroi M., Jung S. M., Okuma M., Shinmyozu K. (1989). A patient with platelets deficient in glycoprotein VI that lack both collagen-induced aggregation and adhesion. *Journal of Clinical Investigation*.

[11] Santoro S. A. (1986). Identification of a 160,000 dalton platelet membrane protein that mediates the initial divalent cation-dependent adhesion of platelets to collagen. *Cell*.

[12] Chen J., López J. A. (2005). Interactions of platelets with subendothelium and endothelium. *Microcirculation*.

[13] Massberg S., Gawaz M., Grüner S. (2003). A crucial role of glycoprotein VI for platelet recruitment to the injured arterial wall in vivo. *Journal of Experimental Medicine*.

[14] Léon C., Hechler B., Freund M. (1999). Defective platelet aggregation and increased resistance to thrombosis in purinergic P2Y1 receptor-null mice. *Journal of Clinical Investigation*.

[15] Cheng Y., Austin S. C., Rocca B. (2002). Role of prostacyclin in the cardiovascular response to thromboxane A2. *Science*.

[16] Kent K. C., Collins L. J., Schwerin F. T., Raychowdhury M. K., Ware J. A. (1993). Identification of functional PGH2/TxA2 receptors on human endothelial cells. *Circulation Research*.

[17] Shattil S. J., Hoxie J. A., Cunningham M., Brass L. F. (1985). Changes in the platelet membrane glycoprotein IIb.IIIa complex during platelet activation. *Journal of Biological Chemistry*.

[18] Gawaz M. (2004). Role of platelets in coronary thrombosis and reperfusion of ischemic myocardium. *Cardiovascular Research*.

[19] Heindl B., Zahler S., Welsch U., Becker B. F. (1998). Disparate effects of adhesion and degranulation of platelets on myocardial and coronary function in postischaemic hearts. *Cardiovascular Research*.

[20] Pachel C., Mathes D., Arias-Loza A.-P. (2016). Inhibition of platelet GPVI protects against myocardial ischemia-reperfusion injury. *Arteriosclerosis, Thrombosis, and Vascular Biology*.

[21] Schüpke S., Hein-Rothweiler R., Mayer K. (2019). Revacept, a novel inhibitor of platelet adhesion, in patients undergoing elective PCI-design and rationale of the randomized ISAR-PLASTER trial. *Thrombosis and Haemostasis*.

[22] Schönberger T., Ziegler M., Borst O. (2012). The dimeric platelet collagen receptor GPVI-Fc reduces platelet adhesion to activated endothelium and preserves myocardial function after transient ischemia in mice. *American Journal of Physiology-Cell Physiology*.

[23] Yang X.-M., Liu Y., Cui L. (2013). Two classes of anti-platelet drugs reduce anatomical infarct size in monkey hearts. *Cardiovascular Drugs and Therapy*.

[24] Heusch G., Gersh B. J. (2017). The pathophysiology of acute myocardial infarction and strategies of protection beyond reperfusion: a continual challenge. *European Heart Journal*.

[25] Turer A. T., Hill J. A. (2010). Pathogenesis of myocardial ischemia-reperfusion injury and rationale for therapy. *The American Journal of Cardiology*.

[26] Ziegler M., Alt K., Paterson B. M. (2016). Highly sensitive detection of minimal cardiac ischemia using positron emission tomography imaging of activated platelets. *Scientific Reports*.

[27] Ziegler M., Wang X., Peter K. (2019). Platelets in cardiac ischaemia/reperfusion injury: a promising therapeutic target. *Cardiovascular Research*.

[28] Schanze N., Bode C., Duerschmied D. (2019). Platelet contributions to myocardial ischemia/reperfusion injury. *Frontiers in Immunology*.

[29] Zuchtriegel G., Uhl B., Puhr-Westerheide D. (2016). Platelets guide leukocytes to their sites of extravasation. *PLOS Biology*.

[30] Barrabés J. A., Garcia-Dorado D., Mirabet M. (2005). Antagonism of selectin function attenuates microvascular platelet deposition and platelet-mediated myocardial injury after transient ischemia. *Journal of the American College of Cardiology*.

[31] Mauler M., Herr N., Schoenichen C. (2019). Platelet serotonin aggravates myocardial ischemia/reperfusion injury via neutrophil degranulation. *Circulation*.

[32] Seligmann C., Prechtl G., Kusus-Seligmann M., Daniel W. G. (2013). A myocardial ischemia- and reperfusion-induced injury is mediated by reactive oxygen species released from blood platelets. *Platelets*.

[33] Mirabet M., Garcia-Dorado D., Inserte J. (2002). Platelets activated by transient coronary occlusion exacerbate ischemia-reperfusion injury in rat hearts. *American Journal of Physiology-Heart and Circulatory Physiology*.

[34] Muessig J. M., Kaya S., Moellhoff L. (2020). A model of blood component-heart interaction in cardiac ischemia-reperfusion injury using a langendorff-based ex vivo assay. *Journal of Cardiovascular Pharmacology and Therapeutics*.

[35] Barrabés J. A., Inserte J., Mirabet M. (2010). Antagonism of P2Y12 or GPIIb/IIIa receptors reduces platelet-mediated myocardial injury after ischaemia and reperfusion in isolated rat hearts. *Thrombosis and Haemostasis*.

[36] Ziegler M., Hohmann J. D., Searle A. K. (2018). A single-chain antibody-CD39 fusion protein targeting activated platelets protects from cardiac ischaemia/reperfusion injury. *European Heart Journal*.

[37] Golebiewska E. M., Poole A. W. (2015). Platelet secretion: from haemostasis to wound healing and beyond. *Blood Reviews*.

[38] Sreeramkumar V., Adrover J. M., Ballesteros I. (2014). Neutrophils scan for activated platelets to initiate inflammation. *Science*.

[39] Rainger G., Chimen M., Harrison M. J. (2015). The role of platelets in the recruitment of leukocytes during vascular disease. *Platelets*.

[40] Brancaleone V., Gobbetti T., Cenac N. (2013). A vasculo-protective circuit centered on lipoxin A4 and aspirin-triggered 15-epi-lipoxin A4 operative in murine microcirculation. *Blood*.

[41] Ying S.-Q., Fang L., Xiang M.-X., Xu G., Shan J., Wang J.-A. (2007). Protective effects of magnesium against ischaemia-reperfusion injury through inhibition of P-selectin in rats. *Clinical and Experimental Pharmacology and Physiology*.

[42] Oostingh G. J., Pozgajova M., Ludwig R. J. (2007). Diminished thrombus formation and alleviation of myocardial infarction and reperfusion injury through antibody- or small-molecule-mediated inhibition of selectin-dependent platelet functions. *Haematologica*.

[43] Babinska A., Azari B., Salifu M. (2007). The F11 receptor (F11R/JAM-A) in atherothrombosis: overexpression of F11R in atherosclerotic plaques. *Thrombosis and Haemostasis*.

[44] Hawrylowicz C. M., Howells G. L., Feldmann M. (1991). Platelet-derived interleukin 1 induces human endothelial adhesion molecule expression and cytokine production. *Journal of Experimental Medicine*.

[45] Muller W. A., Weigl S. A., Deng X., Phillips D. M. (1993). PECAM-1 is required for transendothelial migration of leukocytes. *Journal of Experimental Medicine*.

[46] Sahebkar A., Morris D. R., Biros E., Golledge J. (2013). Association of single nucleotide polymorphisms in the gene encoding platelet endothelial cell adhesion molecule-1 with the risk of myocardial infarction: a systematic review and meta-analysis. *Thrombosis Research*.

[47] Gawaz M. P., Loftus J. C., Bajt M. L., Frojmovic M. M., Plow E. F., Ginsberg M. H. (1991). Ligand bridging mediates integrin alpha IIb beta 3 (platelet GPIIB-IIIA) dependent homotypic and heterotypic cell-cell interactions. *Journal of Clinical Investigation*.

[48] Takagi J., Petre B. M., Walz T., Springer T. A. (2002). Global conformational rearrangements in integrin extracellular domains in outside-in and inside-out signaling. *Cell*.

[49] Weber C., Springer T. A. (1997). Neutrophil accumulation on activated, surface-adherent platelets in flow is mediated by interaction of Mac-1 with fibrinogen bound to alphaIIbbeta3 and stimulated by platelet-activating factor. *Journal of Clinical Investigation*.

[50] Simon D. I., Chen Z., Xu H. (2000). Platelet glycoprotein Ib*α* is a counterreceptor for the leukocyte integrin Mac-1 (CD11b/CD18). *Journal of Experimental Medicine*.

[51] Heinzmann D., Noethel M., von Ungern-Sternberg S. (2020). CD147 is a novel interaction partner of integrin *α*M*β*2 mediating leukocyte and platelet adhesion. *Biomolecules*.

[52] Lefer A. M., Campbell B., Scalia R., Lefer D. J. (1998). Synergism between platelets and neutrophils in provoking cardiac dysfunction after ischemia and reperfusion: role of selectins. *Circulation*.

[53] Seligmann C., Leitsch T., Kusus M. (2003). PMN/Platelets coinfused in Guinea pig hearts exposed to low-flow ischemia have no additive cardiodepressive effect. *Journal of Vascular Research*.

[54] Abdulnour R.-E. E., Dalli J., Colby J. K. (2014). Maresin 1 biosynthesis during platelet-neutrophil interactions is organ-protective. *Proceedings of the National Academy of Sciences*.

[55] Lannan K. L., Spinelli S. L., Blumberg N., Phipps R. P. (2017). Maresin 1 induces a novel pro-resolving phenotype in human platelets. *Journal of Thrombosis and Haemostasis*.

[56] Witte A., Chatterjee M., Lang F., Gawaz M. (2017). Platelets as a novel source of pro-inflammatory chemokine CXCL14. *Cellular Physiology and Biochemistry*.

[57] Mause S. F., Von Hundelshausen P., Zernecke A., Koenen R. R., Weber C. (2005). Platelet microparticles: a transcellular delivery system for RANTES promoting monocyte recruitment on endothelium. *Arteriosclerosis, Thrombosis, and Vascular Biology*.

[58] Chatterjee M., Huang Z., Zhang W. (2011). Distinct platelet packaging, release, and surface expression of proangiogenic and antiangiogenic factors on different platelet stimuli. *Blood*.

[59] Ziff O. J., Bromage D. I., Yellon D. M., Davidson S. M. (2018). Therapeutic strategies utilizing SDF-1*α* in ischaemic cardiomyopathy. *Cardiovascular Research*.

[60] Geisler T., Fekecs L., Wurster T. (2012). Association of platelet-SDF-1 with hemodynamic function and infarct size using cardiac MR in patients with AMI. *European Journal of Radiology*.

[61] Abbott J. D., Huang Y., Liu D., Hickey R., Krause D. S., Giordano F. J. (2004). Stromal cell-derived factor-1*α* plays a critical role in stem cell recruitment to the heart after myocardial infarction but is not sufficient to induce homing in the absence of injury. *Circulation*.

[62] Rath D., Chatterjee M., Holtkamp A. (2016). Evidence of an interaction between TGF-*β*1 and the SDF-1/CXCR4/CXCR7 axis in human platelets. *Thrombosis Research*.

[63] Smith W. B., Noack L., Khew-Goodall Y., Isenmann S., Vadas M. A., Gamble J. R. (1996). Transforming growth factor-beta 1 inhibits the production of IL-8 and the transmigration of neutrophils through activated endothelium. *Journal of Immunology (Baltimore, Md 1950)*.

[64] Hao J., Ju H., Zhao S., Junaid A., Scammell-La Fleur T., Dixon I. M. C. (1999). Elevation of expression of Smads 2, 3, and 4, decorin and TGF-*β* in the chronic phase of myocardial infarct scar healing. *Journal of Molecular and Cellular Cardiology*.

[65] Rath D., Chatterjee M., Müller I. (2014). Platelet expression of transforming growth factor beta 1 is enhanced and associated with cardiovascular prognosis in patients with acute coronary syndrome. *Atherosclerosis*.

[66] Walsh T., Poole A. (2017). Platelets protect cardiomyocytes from ischemic damage. *TH Open*.

[67] Yang B. C., Virmani R., Nichols W. W., Mehta J. L. (1993). Platelets protect against myocardial dysfunction and injury induced by ischemia and reperfusion in isolated rat hearts. *Circulation Research*.

[68] Mehta J. L., Yang B. C., Strates B. S., Mehta P. (1999). Role of TGF-*β*1 in platelet-mediated cardioprotection during ischemia-reperfusion in isolated rat hearts. *Growth Factors*.

[69] Koitabashi N., Danner T., Zaiman A. L. (2011). Pivotal role of cardiomyocyte TGF-*β* signaling in the murine pathological response to sustained pressure overload. *Journal of Clinical Investigation*.

[70] Döring Y., Pawig L., Weber C., Noels H. (2014). The CXCL12/CXCR4 chemokine ligand/receptor axis in cardiovascular disease. *Frontiers in Physiology*.

[71] Urtz N., Gaertner F., Von Bruehl M.-L. (2015). Sphingosine 1-phosphate produced by sphingosine kinase 2 intrinsically controls platelet aggregation in vitro and in vivo. *Circulation Research*.

[72] Davidson S. M., Andreadou I., Barile L. (2019). Circulating blood cells and extracellular vesicles in acute cardioprotection. *Cardiovascular Research*.

[73] Vito C. D., Hadi L. A., Navone S. E. (2016). Platelet-derived sphingosine-1-phosphate and inflammation: from basic mechanisms to clinical implications. *Platelets*.

[74] Jin Z.-Q., Zhou H.-Z., Zhu P. (2002). Cardioprotection mediated by sphingosine-1-phosphate and ganglioside GM-1 in wild-type and PKC*ε* knockout mouse hearts. *American Journal of Physiology-Heart and Circulatory Physiology*.

[75] Jin Z.-Q., Goetzl E. J., Karliner J. S. (2004). Sphingosine kinase activation mediates ischemic preconditioning in murine heart. *Circulation*.

[76] Knapp M. (2011). Cardioprotective role of sphingosine-1-phosphate. *Journal of Physiology and Pharmacology An Official Journal of the Polish Physiological Society*.

[77] Vessey D. A., Li L., Honbo N., Karliner J. S. (2009). Sphingosine 1-phosphate is an important endogenous cardioprotectant released by ischemic pre- and postconditioning. *American Journal of Physiology-Heart and Circulatory Physiology*.

[78] Means C. K., Xiao C.-Y., Li Z. (2007). Sphingosine 1-phosphate S1P2 and S1P3 receptor-mediated Akt activation protects against in vivo myocardial ischemia-reperfusion injury. *American Journal of Physiology-Heart and Circulatory Physiology*.

[79] Egom E. E. A., Mohamed T. M. A., Mamas M. A. (2011). Activation of Pak1/Akt/eNOS signaling following sphingosine-1-phosphate release as part of a mechanism protecting cardiomyocytes against ischemic cell injury. *American Journal of Physiology-Heart and Circulatory Physiology*.

[80] Cohen M. V., Yang X.-M., White J., Yellon D. M., Bell R. M., Downey J. M. (2016). Cangrelor-mediated cardioprotection requires platelets and sphingosine phosphorylation. *Cardiovascular Drugs and Therapy*.

[81] Russo I., Femminò S., Barale C. (2018). Cardioprotective properties of human platelets are lost in uncontrolled diabetes mellitus: a study in isolated rat hearts. *Frontiers in Physiology*.

[82] Jung J. H., Tantry U. S., Gurbel P. A., Jeong Y.-H. (2015). Current antiplatelet treatment strategy in patients with diabetes mellitus. *Diabetes & Metabolism Journal*.

[83] Evangelou A. M. (1994). Platelet-activating factor (PAF): implications for coronary heart and vascular diseases. *Prostaglandins, Leukotrienes and Essential Fatty Acids*.

[84] Bussolino F., Breviario F., Tetta C., Aglietta M., Mantovani A., Dejana E. (1986). Interleukin 1 stimulates platelet-activating factor production in cultured human endothelial cells. *Journal of Clinical Investigation*.

[85] Penna C., Bassino E., Alloatti G. (2011). Platelet activating factor: the good and the bad in the ischemic/reperfused heart. *Experimental Biology and Medicine*.

[86] Penna C., Alloatti G., Cappello S. (2005). Platelet-activating factor induces cardioprotection in isolated rat heart akin to ischemic preconditioning: role of phosphoinositide 3-kinase and protein kinase C activation. *American Journal of Physiology-Heart and Circulatory Physiology*.

[87] Montrucchio G., Alloatti G., Mariano F (1990). Role of platelet-activating factor in the reperfusion injury of rabbit ischemic heart. *The American Journal of Pathology*.

[88] Ko W., Lang D., Hawes A. S., Zelano J. A., Isom O. W., Krieger K. H. (1993). Platelet-activating factor antagonism attenuates platelet and neutrophil activation and reduces myocardial injury during coronary reperfusion. *Journal of Surgical Research*.

[89] Brill A., Dashevsky O., Rivo J., Gozal Y., Varon D. (2005). Platelet-derived microparticles induce angiogenesis and stimulate post-ischemic revascularization. *Cardiovascular Research*.

[90] Zhang W., Chen C., Wang J., Liu L., He Y., Chen Q. (2018). Mitophagy in cardiomyocytes and in platelets: a major mechanism of cardioprotection against ischemia/reperfusion injury. *Physiology*.

[91] Zhang W., Ren H., Xu C. (2016). Hypoxic mitophagy regulates mitochondrial quality and platelet activation and determines severity of I/R heart injury. *Elife*.

[92] Bosetti F., Brizzi F., Barogi S. (2002). Cytochrome c oxidase and mitochondrial F1F0-ATPase (ATP synthase) activities in platelets and brain from patients with Alzheimer’s disease. *Neurobiology of Aging*.

[93] Kramer P. A., Ravi S., Chacko B., Johnson M. S., Darley-Usmar V. M. (2014). A review of the mitochondrial and glycolytic metabolism in human platelets and leukocytes: implications for their use as bioenergetic biomarkers. *Redox Biology*.

[94] Liu L., Feng D., Chen G. (2012). Mitochondrial outer-membrane protein FUNDC1 mediates hypoxia-induced mitophagy in mammalian cells. *Nature Cell Biology*.

[95] Zhang W., Siraj S., Zhang R., Chen Q. (2017). Mitophagy receptor FUNDC1 regulates mitochondrial homeostasis and protects the heart from I/R injury. *Autophagy*.

[96] Zhou H., Li D., Zhu P. (2017). Melatonin suppresses platelet activation and function against cardiac ischemia/reperfusion injury via PPAR*γ*/FUNDC1/mitophagy pathways. *Journal of Pineal Research*.

[97] Théry C., Witwer K. W., Aikawa E. (2018). Minimal information for studies of extracellular vesicles 2018 (MISEV2018): a position statement of the International Society for Extracellular Vesicles and update of the MISEV2014 guidelines. *Journal of Extracellular Vesicles*.

[98] George M., Ganesh M. R., Sridhar A. (2015). Evaluation of endothelial and platelet derived microparticles in patients with acute coronary syndrome. *Journal of Clinical and Diagnostic Research*.

[99] Finkielsztein A., Mascarenhas L., Butin-Israeli V., Sumagin R. (2018). Isolation and characterization of neutrophil-derived microparticles for functional studies. *Journal of Visualized Experiments*.

[100] Olatunya O. S., Lanaro C., Longhini A. L. (2019). Red blood cells microparticles are associated with hemolysis markers and may contribute to clinical events among sickle cell disease patients. *Annals of Hematology*.

[101] Femminò S., Penna C., Margarita S., Comità S., Brizzi M. F., Pagliaro P. (2020). Extracellular vesicles and cardiovascular system: biomarkers and cardioprotective effectors. *Vascular Pharmacology*.

[102] Zaldivia M. T. K., McFadyen J. D., Lim B., Wang X., Peter K. (2017). Platelet-derived microvesicles in cardiovascular diseases. *Frontiers in Cardiovascular Medicine*.

[103] Nieuwland R., Berckmans R. J., Rotteveel-Eijkman R. C. (1997). Cell-derived microparticles generated in patients during cardiopulmonary bypass are highly procoagulant. *Circulation*.

[104] Barry O. P., Praticò D., Savani R. C., FitzGerald G. A. (1998). Modulation of monocyte-endothelial cell interactions by platelet microparticles. *Journal of Clinical Investigation*.

[105] Jung C., Sörensson P., Saleh N., Arheden H., Rydén L., Pernow J. (2012). Circulating endothelial and platelet derived microparticles reflect the size of myocardium at risk in patients with ST-elevation myocardial infarction. *Atherosclerosis*.

[106] Vélez P., Parguiña A., Ocaranza-Sánchez R. (2014). Identification of a circulating microvesicle protein network involved in ST-elevation myocardial infarction. *Thrombosis and Haemostasis*.

[107] Porto I., Biasucci L. M., De Maria G. L. (2012). Intracoronary microparticles and microvascular obstruction in patients with ST elevation myocardial infarction undergoing primary percutaneous intervention. *European Heart Journal*.

[108] Christersson C., Thulin Å., Siegbahn A. (2017). Microparticles during long-term follow-up after acute myocardial infarction: association to atherosclerotic burden and risk of cardiovascular events. *Thrombosis and Haemostasis*.

[109] Ma F., Liu H., Shen Y., Zhang Y., Pan S. (2015). Platelet-derived microvesicles are involved in cardio-protective effects of remote preconditioning. *International Journal of Clinical and Experimental Pathology*.

[110] Liu M., Wang Y., Zhu Q. (2018). Protective effects of circulating microvesicles derived from ischemic preconditioning on myocardial ischemia/reperfusion injury in rats by inhibiting endoplasmic reticulum stress. *Apoptosis*.

[111] García Á. (2016). Platelet clinical proteomics: facts, challenges, and future perspectives. *Proteomics-Clinical Applications*.

[112] Cheow E. S. H., Cheng W. C., Lee C. N., De Kleijn D., Sorokin V., Sze S. K. (2016). Plasma-derived extracellular vesicles contain predictive biomarkers and potential therapeutic targets for Myocardial Ischemic (MI) injury. *Molecular & Cellular Proteomics*.

[113] Vélez P., García Á. (2015). Platelet proteomics in cardiovascular diseases. *Translational Proteomics*.

[114] Raphael R., Purushotham D., Gastonguay C. (2016). Combining patient proteomics and in vitro cardiomyocyte phenotype testing to identify potential mediators of heart failure with preserved ejection fraction. *Journal of Translational Medicine*.

[115] Penna C., Femminò S., Alloatti G., Brizzi M. F., Angelone T., Pagliaro P. (2021). Extracellular vesicles in comorbidities associated with ischaemic heart disease: focus on sex, an overlooked factor. *Journal of Clinical Medicine*.

[116] Sluijter J. P. G., Davidson S. M., Boulanger C. M. (2018). Extracellular vesicles in diagnostics and therapy of the ischaemic heart: position paper from the working group on cellular biology of the heart of the European society of cardiology. *Cardiovascular Research*.

[117] Stepanian A., Bourguignat L., Hennou S. (2013). Microparticle increase in severe obesity: not related to metabolic syndrome and unchanged after massive weight loss. *Obesity*.

[118] Bei Y., Xu T., Lv D. (2017). Exercise-induced circulating extracellular vesicles protect against cardiac ischemia-reperfusion injury. *Basic Research in Cardiology*.

[119] Murakami T., Horigome H., Tanaka K. (2007). Impact of weight reduction on production of platelet-derived microparticles and fibrinolytic parameters in obesity. *Thrombosis Research*.

[120] Campello E., Zabeo E., Radu C. M. (2016). Dynamics of circulating microparticles in obesity after weight loss. *Internal and Emergency Medicine*.

[121] Rigamonti A. E., Bollati V., Pergoli L. (2020). Effects of an acute bout of exercise on circulating extracellular vesicles: tissue-, sex-, and BMI-related differences. *International Journal of Obesity*.

[122] Wilhelm E. N., González-Alonso J., Parris C., Rakobowchuk M. (2016). Exercise intensity modulates the appearance of circulating microvesicles with proangiogenic potential upon endothelial cells. *American Journal of Physiology-Heart and Circulatory Physiology*.

[123] Penna C., Femminò S., Tapparo M. (2020). The inflammatory cytokine IL-3 hampers cardioprotection mediated by endothelial cell-derived extracellular vesicles possibly via their protein cargo. *Cells*.

[124] Preston R. A., Jy W., Jimenez J. J. (2003). Effects of severe hypertension on endothelial and platelet microparticles. *Hypertension*.

[125] Agouni A., Lagrue-Lak-Hal A. H., Ducluzeau P. H. (2008). Endothelial dysfunction caused by circulating microparticles from patients with metabolic syndrome. *The American Journal of Pathology*.

[126] Amosse J., Durcin M., Malloci M. (2018). Phenotyping of circulating extracellular vesicles (EVs) in obesity identifies large EVs as functional conveyors of Macrophage Migration Inhibitory Factor. *Molecular Metabolism*.

[127] Varga Z. V., Kupai K., Szűcs G. (2013). MicroRNA-25-dependent up-regulation of NADPH oxidase 4 (NOX4) mediates hypercholesterolemia-induced oxidative/nitrative stress and subsequent dysfunction in the heart. *Journal of Molecular and Cellular Cardiology*.

[128] Perrino C., Barabási A.-L., Condorelli G. (2017). Epigenomic and transcriptomic approaches in the post-genomic era: path to novel targets for diagnosis and therapy of the ischaemic heart? Position paper of the European society of cardiology working group on cellular biology of the heart. *Cardiovascular Research*.

[129] Sabatier F., Darmon P., Hugel B. (2002). Type 1 and type 2 diabetic patients display different patterns of cellular microparticles. *Diabetes*.

[130] Salem M. A. E. K., Adly A. A. M., Ismail E. A. R., Darwish Y. W., Kamel H. A. (2015). Platelets microparticles as a link between micro- and macro-angiopathy in young patients with type 1 diabetes. *Platelets*.

[131] Zhang X., McGeoch S. C., Johnstone A. M. (2014). Platelet-derived microparticle count and surface molecule expression differ between subjects with and without type 2 diabetes, independently of obesity status. *Journal of Thrombosis and Thrombolysis*.

[132] Chiva-Blanch G., Suades R., Padró T. (2016). Microparticle shedding by erythrocytes, monocytes and vascular smooth muscular cells is reduced by aspirin in diabetic patients. *Revista Española de Cardiología (English Edition)*.

[133] Davidson S. M., Riquelme J. A., Takov K. (2018). Cardioprotection mediated by exosomes is impaired in the setting of type II diabetes but can be rescued by the use of non-diabetic exosomes in vitro. *Journal of Cellular and Molecular Medicine*.

[134] Mastrocola R., Aragno M., Alloatti G., Collino M., Penna C., Pagliaro P. (2018). Metaflammation: tissue-specific alterations of the NLRP3 inflammasome platform in metabolic syndrome. *Current Medicinal Chemistry*.

[135] Mastrocola R., Collino M., Penna C. (2016). Maladaptive modulations of nlrp3 inflammasome and cardioprotective pathways are involved in diet-induced exacerbation of myocardial ischemia/reperfusion injury in mice. *Oxidative Medicine and Cellular Longevity*.

[136] Birnbaum Y., Birnbaum G. D., Birnbaum I., Nylander S., Ye Y. (2016). Ticagrelor and rosuvastatin have additive cardioprotective effects via adenosine. *Cardiovascular Drugs and Therapy*.

[137] Mastrocola R., Penna C., Tullio F. (2016). Pharmacological inhibition of NLRP3 inflammasome attenuates myocardial ischemia/reperfusion injury by activation of RISK and mitochondrial pathways. *Oxidative Medicine and Cellular Longevity*.

[138] Qiao J., Wu X., Luo Q. (2018). NLRP3 regulates platelet integrin *α*IIb*β*3 outside-in signaling, hemostasis and arterial thrombosis. *Haematologica*.

[139] Peng H., Wu H., Zhang G. (2020). Expression and clinical prognostic value of platelet NLRP3 in acute coronary syndrome. *International Journal of General Medicine*.

[140] Penna C., Aragno M., Cento A. S. (2020). Ticagrelor conditioning effects are not additive to cardioprotection induced by direct NLRP3 inflammasome inhibition: role of RISK, NLRP3, and redox cascades. *Oxidative Medicine and Cellular Longevity*.

[141] Baigent C., Sudlow C., Collins R., Peto R. (2002). Collaborative meta-analysis of randomised trials of antiplatelet therapy for prevention of death, myocardial infarction, and stroke in high risk patients. *The BMJ*.

[142] Abergel E., Nikolsky E. T. (2010). An investigational oral antiplatelet treatment for reduction of major adverse cardiac events in patients with acute coronary syndrome. *Vascular Health and Risk Management*.

[143] Kupatt C., Wichels R., Horstkotte J., Krombach F., Habazettl H., Boekstegers P. (2002). Molecular mechanisms of platelet-mediated leukocyte recruitment during myocardial reperfusion. *Journal of Leukocyte Biology*.

[144] Ridker P. M., Cushman M., Stampfer M. J., Tracy R. P., Hennekens C. H. (1997). Inflammation, aspirin, and the risk of cardiovascular disease in apparently healthy men. *New England Journal of Medicine*.

[145] Husain S., Andrews N. P., Mulcahy D., Panza J. A., Quyyumi A. A. (1998). Aspirin improves endothelial dysfunction in atherosclerosis. *Circulation*.

[146] Yang X.-M., Liu Y., Cui L. (2013). Platelet P2Y12 blockers confer direct postconditioning-like protection in reperfused rabbit hearts. *Journal of Cardiovascular Pharmacology and Therapeutics*.

[147] Vilahur G., Gutiérrez M., Casani L. (2018). P2Y12 antagonists and cardiac repair post-myocardial infarction: global and regional heart function analysis and molecular assessments in pigs. *Cardiovascular Research*.

[148] Montalescot G., Wiviott S. D., Braunwald E. (2009). Prasugrel compared with clopidogrel in patients undergoing percutaneous coronary intervention for ST-elevation myocardial infarction (TRITON-TIMI 38): double-blind, randomised controlled trial. *The Lancet*.

[149] Wang K., Zhou X., Huang Y (2010). Adjunctive treatment with ticagrelor, but not clopidogrel, added to tPA enables sustained coronary artery recanalisation with recovery of myocardium perfusion in a canine coronary thrombosis model. *Thrombosis and Haemostasis*.

[150] Roubille F., Lairez O., Mewton N. (2012). Cardioprotection by clopidogrel in acute ST-elevated myocardial infarction patients: a retrospective analysis. *Basic Research in Cardiology*.

[151] Feliu C., Peyret H., Brassart-Pasco S. (2020). Ticagrelor prevents endothelial cell apoptosis through the adenosine signalling pathway in the early stages of hypoxia. *Biomolecules*.

